# Phylogenomics of *Rhodocyclales* and its distribution in wastewater treatment systems

**DOI:** 10.1038/s41598-020-60723-x

**Published:** 2020-03-03

**Authors:** Zhongjie Wang, Wenqing Li, Hao Li, Wei Zheng, Feng Guo

**Affiliations:** 1 School of Life Sciences, Xiamen University, Fujian, 361102 P.R. China; 2 College of The Environments and Ecology, Xiamen University, Fujian, 361102 P.R. China

**Keywords:** Microbial ecology, Water microbiology

## Abstract

*Rhodocyclales* is an abundant bacterial order in wastewater treatment systems and putatively plays key roles in multiple functions. Its phylogenomics, prevalence of denitrifying genes in sub-lineages and distribution in wastewater treatment plants (WWTPs) worldwide have not been well characterized. In the present study, we collected 78 *Rhodocyclales* genomes, including 17 from type strains, non-type strains and genome bins contributed by this study. Phylogenomics indicated that the order could be divided into five family-level lineages. With only a few exceptions (mostly in *Rhodocyclaceae*), *nirS*-containing genomes in this order usually contained the downstream genes of *norB* and *nosZ*. Multicopy of denitrifying genes occurred frequently and events of within-order horizontal transfer of denitrifying genes were phylogenetically deduced. The distribution of *Rhodocyclaceae*, *Zoogloeaceae* and *Azonexaceae* in global WWTPs were significantly governed by temperature, mixed liquor suspended solids, *etc*. Metagenomic survey showed that the order generally ranked at the top or second for different denitrifying genes in wastewater treatment systems. Our results provided comprehensive genomic insights into the phylogeny and features of denitrifying genes of *Rhodocyclales*. Its contribution to the denitrifying gene pool in WWTPs was proved.

## Introduction

Activated sludge, which is a widely utilized biological process for the treatment of municipal and industrial wastewaters around the world for over a century^1,2^, relies on a complex consortium of microorganisms to remove pollutants and facilitate separation of flocs and water^3^. A key functional microbial taxon in wastewater treatment systems is *Rhodocyclales*, which is dominant in activated sludge samples according to the relative abundance of 16S rRNA genes and hybridization approach^4,5^. Diverse genera in *Rhodocyclales* can perform special treating functions, such as *Candidatus* Accumulibacter (*Accumulibacter* hereinafter) in enhanced biological phosphorus removal (EBPR) bioreactor in which the process of removing phosphorus is enhanced under the alternating appearance of anaerobic and aerobic conditions^6^, *Zoogloea* in formation of bioflocs^7^ and *Azoarcus* and *Thauera* in the degradation of polycyclic aromatic hydrocarbon^8,9^. Many species in this order contributed to denitrification, as depicted by stable isotope probing and fluorescence *in situ* hybridization coupled with microautoradiography studies^10–12^.

The order *Rhodocyclales*, which is affiliated with *Betaproteobacteria*, previously consisted of a single family *Rhodocyclaceae*^13^. Recently, the order is reclassified into three families, namely, *Azonexaceae*, *Rhodocyclaceae* and *Zoogloeaceae* and one undecided group, that is, *Azovibrio_f*, on the basis of the phylogenetic analysis of 16S rRNA gene sequences and physiological traits^14^. Phylogenomic taxonomy based on multiple core genes has exhibited its superiority in prokaryotic classification across domain to species levels^15,16^ and in illuminating the evolutionary adaption^17^. To date, *Rhodocyclales* have not been well characterized phylogenomically. This inadequacy hindered systematic understanding on their ecological distribution and functions, especially in wastewater treatment systems.

The key function of many *Rhodocyclales* species in wastewater treatment plants (WWTPs) is thought to be denitrification. However, to our knowledge, no comprehensive studies concerning the abundance and diversity of denitrifying genes in the activated sludge from sewage treatment plants have been conducted, except for few case reports^18,19^. Rapid gain and loss of certain denitrification genes have been deciphered across many bacterial lineages^20,21^. Closely related bacteria do not necessarily conserve the denitrifying capabilities^22^, while closely related genes have been discovered in distantly related bacteria and vice versa^23^. To date, the distribution and potential horizontal gene transfer (HGT) of denitrifying genes in *Rhodocyclales* have not been comprehensively characterized. A previous rRNA-based survey has revealed that the relative abundance of the order is usually high in activated sludge samples. However, its corresponding contribution to denitrification in wastewater treatment is unclear.

In this study, we contributed 17 high- or moderate-quality genomes of *Rhodocyclales*, including three from type strains, four from isolates and ten bins from metagenomes. A total of 78 genomes of *Rhodocyclales* were used to conduct phylogenetic analysis to improve our understanding of the classification and denitrification gene distribution within the order. By referring to an extensive survey of bacterial community and environmental parameters on global WWTPs^24^, the factors governing the distribution of *Rhodocyclales* could be revealed. Metagenomic mining on the distribution of denitrifying genes was also performed to examine the relative abundance of *Rhodocyclales*-derived denitrification genes in activated sludge samples. Our study, therefore, aimed to reveal the phylogenomics of *Rhodocyclales* and the distribution and potential weight of denitrification of the order in wastewater treatment.

## Materials and Methods

### Sources of 16S rRNA gene sequences, genomic data and strains

A total of 99 genomes of *Rhodocyclales* were collected in this study. Among them, 82 genomes were downloaded from NCBI and IMG website (including genome and assembly by searching using keyword “*Rhodocyclales*”). Three strains of DSM109 (*Rhodocyclus tenuis*), DSM6832 (*Propionivibrio limicola*) and DSM15637 (*Dechloromonas hortensis*) were purchased from DSMZ. Four isolates (named with *_Iso) were isolated from the activated sludge of a full-scale WWTP (located in Xiamen, China) on the R2A medium. We also sequenced 4 metagenomes, that is, 2 from the WWTP above and 2 from laboratory-scale EBPR bioreactors, from which 10 draft genomes of *Rhodocyclales* (named with *_Bin) were extracted via genomic binning (see the following parts for details). However, 9 genomes from the databases were removed due to low completeness and high contamination and the remaining 90 genomes (73 downloaded genomes and 17 contributed by the present study) were used for further study. Table S1 lists the information of genomes.

One-hundred and twenty species-level representatives (including type strains and clone sequences) of 16S rRNA gene sequences of *Rhodocyclales* were downloaded from EzBioCloud^25^. These sequences were filtrated to 108 by de-redundancy (>99% similarity). The 25 other sequences were extracted from the above-mentioned genomes (if they exist) using RNAmmer^26^. We also obtained 16S rRNA gene seqeuences from XMAS_Iso1–4 and DSM15637. Five sequences from *Nitrosomonadales* were used as the outgroup.

### Metagenomes and genomes contributed by this study

For the four metagenomes contributed by the present study, AS samples were fixed with ethanol (1:1 in volume) on site and stored at −20 °C until DNA extraction. During DNA extraction, biomass was collected by centrifugation. Then, DNA was extracted using the FastDNA^TM^ Spin Kit for Soil (MP Biomedicals, US) in accordance with the instruction manual. For DNA from isolates and AS samples, Illumina Truseq DNA PCR-free (insert fragment of 350 bp) libraries were constructed before paired-end sequencing on the Illumina HiSeq X10 platform (2 × 150 bp).

The genomes of isolates were assembled with SPAdes with the commands careful–mismatch–correction with the kmer lengths of 55, 77, 99 and 127^27^. Assembly was conducted using Megahit with the default parameters^28^. Contigs shorter than 1 kb were removed from downstream analysis. The genomes of bins were manually extracted from metagenomes assembly using R package mmgenome based on the differential coverage principle^29,30^. Bowtie2 and Sam-tools were used to map, sort and estimate the read depth of the resulting files^31,32^. Completeness and contamination of genomes were estimated using CheckM^33^. Genomes with completeness higher than 70% and contamination less than 10% were retained for further analysis.

### Construction of phylogenetic tree based on 16S rRNA gene and genomes

All 16S rRNA gene sequences were aligned using MAFFT with the options -maxiterate 1000 -localpair^34^. The aligned sequences were then used to reconstruct a maximum-likelihood (ML) tree using RAxML under the optimal general time-reversible model and GAMMA distribution determined as the best model by MEGA7^35,36^. RAxML searched for the best-scoring ML tree after generating 500 rapid bootstrap replicates. We also constructed neighbor-joining (NJ) and maximum parsimony (MP) method phylogenetic trees in MEGA7 and Bayesian (BY) phylogenetic tree using MrBayes with the Mkv model and gamma-distributed rate variation^37^ to verify the topology and support values of the ML tree.

A set of 40 ubiquitously conserved single-copy marker genes was extracted from the genomes using BLASTP to construct a robust phylogeny for phylogenomic analysis^38^. For a given genome, if any single-copy marker gene had multiple copies, then all copies of that gene were excluded for the tree construction. These genes were aligned using the same options above in MAFFT. Subsequently, the aligned protein sequences were concatenated into a continuous alignment using Gblocks^39^. Then, a phylogenetic analysis was performed using RAxML on the concatenated 40 aligned conserved marker genes with a model of PROTGAMMAAUTO using RAxML with 500 bootstraps.

### Genome annotation

Open reading frames (ORFs) were predicted using Prodigal [34]. Automatic annotation of genomes was performed using Prokka^40^. Information of genes or ORFs related to several functions, such as anoxygenic photoheterotrophy (*pufL* and *pufM*), nitrogen fixation (*nifA*, *nifB*, *nifL*, *nifD* and *nifK*), chlorate, perchlorate and selenate respiration (chlorate reductase, perchlorate reductase and selenite reductase), detoxification to reactive oxygen species (superoxide dismutase catalase and peroxidase) and denitrification (*narG*, *nirS*, *nirK*, *norB* and *nosZ*), were obtained from the annotation result. All the ORFs underwent online BLASTP against NR database to validate the congruence of the function. All denitrifying genes except for *nirK* (because of its low frequency) were used to conduct orthologous gene clustering using MCL with an optimal inflation value of 2^41^.

### RDA analysis of the distribution of *Rhodocyclales* in WWTPs

The distribution of *Rhodocyclales* in global WWTPs was referred to^24^, which covered 273 WWTPs. Only 131 plants with less than five out of ten picked environmental variables being NA would be kept. The 10 variables were mixed liquor suspended solids (MLSS), latitude, year of running, mixed liquid temperature, percentage of industrial wastewater, chemical oxygen demand, dissolved oxygen, pH, ammonia nitrogen, total phosphorus and biochemical oxygen demand in AS tanks. We randomly selected one of the replications for each plant. The OTUs were annotated using EZBioCloud database^25^ and Mothur^42^ and the relative abundance of *Rhodocyclales* at order, family and OTU levels were calculated on the basis of the reference-provided OTU table and the above-mentioned annotation. Redundancy analysis (RDA) was conducted using vegan R package to study the relationship between the distribution of sublineages of *Rhodocyclales* and environmental variables^43^.

### Construction of the database of denitrifying genes

A customized database of the four denitrifying genes (*narG*, *nirS*, *norB* and *nosZ*) that covered major bacterial lineages was constructed to survey the contribution of *Rhodocyclales* denitrifying genes in AS metagenomes. We downloaded all denitrifying genes amino acid sequences from the RefSeq database using Entrez^44^. Denitrifying genes from *Rhodocyclales* that were extracted from the genomes were added to the database. We re-annotated the above-mentioned sequences using EggNOG^45^ under DIAMOND^46^ and HMMER^47^ to filter false denitrifying gene sequences. The congruently annotated sequences for both methods were kept as the database of denitrifying genes.

The phylogenetic tree of *nirS* across the database covering lineages was built as the key step of denitrification and due to its wide distribution in *Rhodocyclales*. The comparison of all genes was conducted using BLASTP with the command e-value 1e-5 to remove redundant genes. The BLAST result was filtered under the percent identity of 70% and the query coverage of 75%^48^. All *nirS* sequences underwent orthologous gene clustering using MCL with an inflation value of 2^41^. For each cluster, the longest representative of each taxonomic family was kept. The remaining 217 sequences were used to construct a phylogenetic tree in RAxML by applying PROTGAMMAAUTO and 200 bootstraps. Poorly aligned internal alignment regions were deleted using Gblocks to guarantee less 50% gaps in a given position. NJ trees of *narG*, *nirS*, *norB* and *nosZ* from the *Rhodocyclales* were constructed with MEGA7 to detect the potential gene horizontal transfer within the order.

### Distribution of denitrifying genes in AS metagenomes

Forty-four metagenomes from activated sludge (8 from laboratory bioreactors and 36 from globally collected full-scale samples) were included in the analysis (Table S2). The samples contained four contributed by this study and 40 samples download from MG-RAST database^49^. They were aligned to the constructed denitrifying gene databases using DIAMOND with the parameters query-cover 70 -id 50 -max-target-seqs. 1 -evalue 1e-5 -sensitive. Two hundred hits were randomly subsampled and checked for false-positive results via online BLASTX against the NR database to determine the optimal similarity cutoff for each denitrifying gene. Figure S1 shows the false-positive rates under various identity cutoffs and the fraction of various similarities. A cutoff of 70% similarity was proposed. The number of 16S rRNA gene reads was determined by SortMeRNA for data normalization^50^. The visualization was performed using R package pheatmap^51^.

### Sequence data availability

Five sequences of 16S rRNA of *Rhodocyclales* for cloning library construction have been deposited in GenBank under accession no. MK554699-MK554703. The 17 genomes contributed in this study have been deposited at DDBJ/ENA/GenBank under the accession nos. SSSP00000000-SSSZ00000000, SSTA00000000-SSTB00000000 and SSXU00000000-SSXX00000000. The Illumina metagenomic datasets are available in the NCBI Sequence Read Archive database under the BioProject accession no. PRJNA524249.

## Results and Discussion

### Phylogeny of *Rhodocyclales* based on 16S rRNA gene

**Figure 1:**
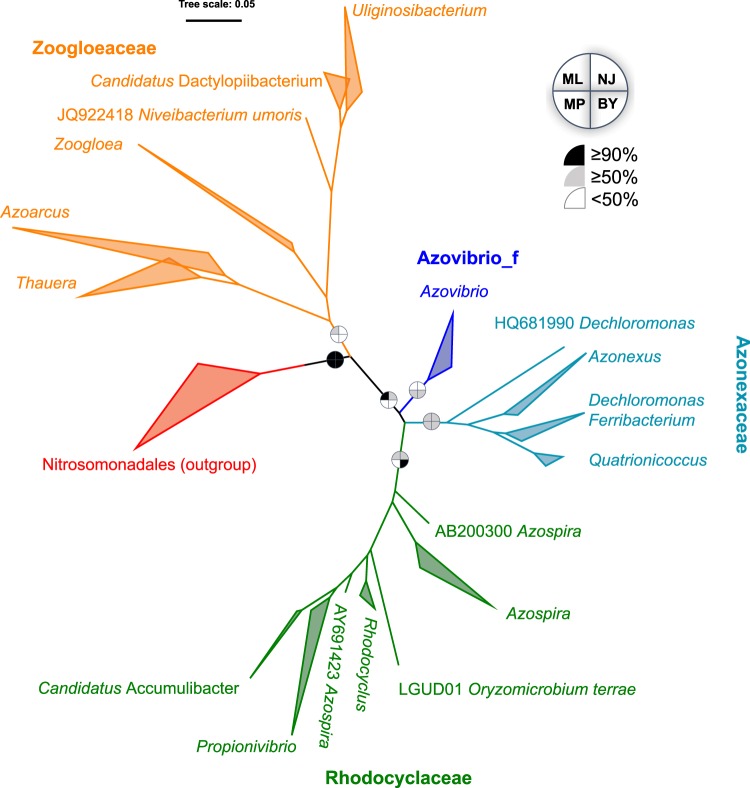
Maximum-likelihood tree on the basis of the 16S rRNA gene of *Rhodocyclales*. The bootstrap values on nodes indicating the percentage of reconstructions in which the topology was preserved using the maximum-likelihood (ML), neighbor-joining (NJ), maximum-parsimony (MP) and Bayesian (BY) methods. Node values are Bayesian posterior probability support values. Scale bar represents substitutions/site. Colored branches and labels indicate major clades. The outgroup contained sequences from *Nitrosomonadales*.

Consistent with the existing classification result only based on type strains^14^, phylogenetic analysis on the basis of 16S rRNA gene sequences supported that the order consisted of four family-level lineages, namely, *Rhodocyclaceae*, *Azonexaceae*, *Zoogloeaceae* and *Azovibrio_f* (Figs. 1 and S2). *Zoogloeaceae* exhibited a considerably higher intra-lineage diversity and phylogenetic depth than the other lineages. *Rhodocyclaceae* and *Azonexaceae* were moderately supported with bootstrapping values over 50% for at least three methods (ML, NJ and BY). However, the two other lineages were only weakly supported by one or two methods. As expected, this result based on the rRNA gene provided an ambiguous classification, as pointed out by previous studies^52,53^. By contrast, the genus-level phylogeny was consistent with the current taxonomic system in most cases, except for several sequences (not from type strains) that were previously defined as *Dechloromonas* and *Azospira*.

### Five family-level sub-lineages in *Rhodocyclacles* indicated by phylogenomics

**Figure 2:**
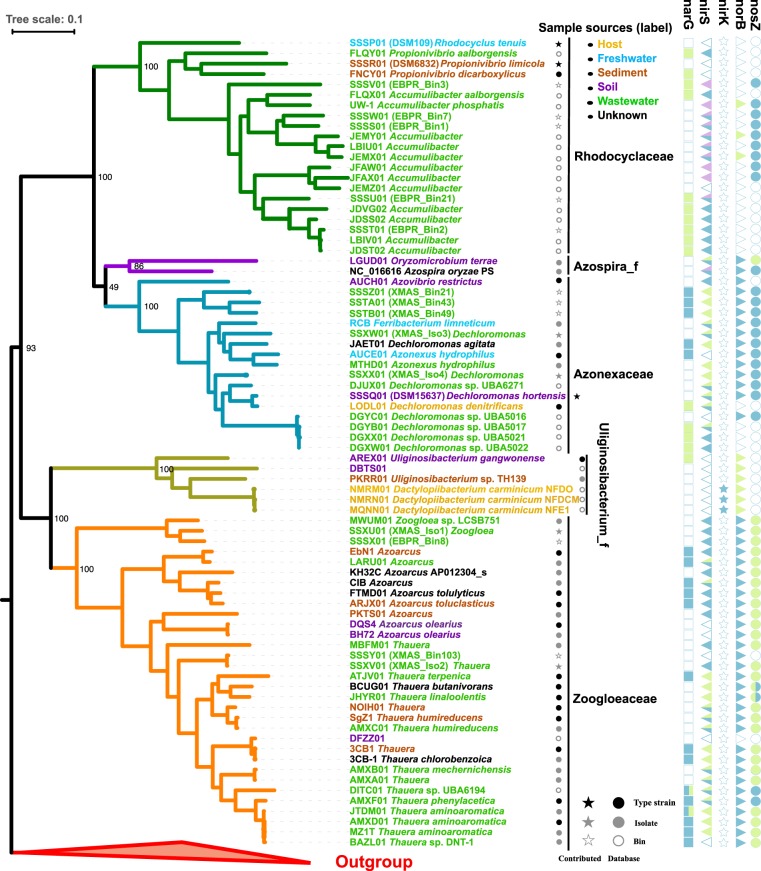
Maximum-likelihood tree on the basis of 40 concatenated universal genes. Distinctive colors of branches stand for different family-level lineages and outgroups. The colors in the labels stand for sample sources as annotated. The numbers on the side of the nodes show the bootstrap value (n = 500). The right side shows the distribution of five denitrifying genes in *Rhodocyclales*, with each shape representing a gene. Blank indicates absence and different colors in the shape represent different gene clusters for a given gene.

As shown in Fig. 2, the phylogenomic tree contained 90 predefined *Rhodocyclales* genomes and two genomes from *Nitrosomonadales* as outgroups (*Sulfuritalea hydrogenivorans* and *Methyloversatilis discipulorum*, which were previously incorrectly assigned in *Rhodocyclales*^13^). It was very clear that this phylogenomic tree had a higher resolution and provided more robust and accurate phylogenetic relationships (Fig. 2). Twelve genomes were incorrectly classified because they were clustered with the outgroups from *Nitrosomonadales*. As for the phylogeny of the remaining 78 genomes, we proposed that the order should be classified from existing three family-level lineages into five family-level lineages, namely, *Rhodocyclaceae*, *Azonexaceae*, *Zoogloeaceae*, *Azospira_f,* and *Uliginosibacterium_f*, with the support of high BS value for each lineage. The classification of the first three families was generally consistent with the phylogenetic analysis on the basis of 16S rRNA gene sequences, whereas the two latter families were not. Containing only two genomes of *Azospira oryzae* and *Oryzomicrobium terrae*, *Azospira_f* that was classified as a member of *Rhodocyclaceae* based on 16S rRNA gene^14^ (and Fig. 1) was phylogenomically highly related to *Azonexaceae*. However, the weakly supported node (BS = 49%) between *Azospira_f* and *Azonexaceae* suggested that the former could be designated as a family-level lineage independently. For the proposed *Uliginosibacterium_f* that contained two genera (*Uliginosibacterium* and *Candidatus* Dactylopiibacterium), the rRNA-based phylogeny also supported that the two genera, together with *Niveibacterium umoris*, formed a clade separated from *Zoogloea* (Fig. 1). The phylogenomic affiliation of this genus was unclear because of the absence of genomes from *Niveibacterium*. The only one genome from *Azovibrio restrictus* (the type species of the genus) presented as a single branch and clustered with the other taxa within *Azonexaceae*. Thus, we still designated this genome as a member of *Azonexaceae* in this study. The determination of the taxonomic rank of this clade should require additional related genomes and information. Polyphyletic distribution was observed for type strains in *Azoarcus* (e.g., DQS4 and ARJX01). Therefore, further clarification should be conducted for the genera.

Strains in *Azospira_f* and *Uliginosibacterium_f* were obtained from soil, sediment and host-associated environments and no genomes came from wastewater-related samples. However, *Azospira* (if correctly classified) has been detected in many AS samples by high-throughput sequencing of the rRNA gene^5^. The three other lineages, namely, *Rhodocyclaceae*, *Azonexaceae* and *Zoogloeacea*, contained a high proportion of strains from activated sludge. However, the phenomenon should be attributed to the biased contribution of wastewater-related strains in the total genome dataset. The wastewater-related strain as an artificial system must be sourced from other natural environments. Soil and sediment could be the potential sources because the wastewater strains usually have phylogenetically related taxa in these environments (Fig. 2).

### Distribution of genes encoding functional traits related to taxonomy

A set of key genes corresponding to phenotypic classification of this order was sought in the genomes. The features included anoxygenic photoheterotrophy, nitrogen fixation, utilization of chlorate, perchlorate and selenate as electron acceptors and reactive oxygen species detoxification^54^, (Table 1). Species of *Rhodocyclus*, which is a genus of *Rhodocyclales*, are anoxygenic photoheterotrophic organisms. Despite the absence of genome from the species *R. purpureus*, the key genes *pufM* and *pufL* were only detected in this genus. The phylogenetic topology in this order (Fig. 2) indicated that this metabolic capability should likely be horizontally gained for this genus (less likely lost in all other clades). Moreover, superoxide dismutase and catalase-peroxidase detected in nearly all genomes suggested the aerobic or aerotolerant property in the majority of this order^55^. The occasional absence of the two enzymes might be due to the genomic incompleteness. Nitrogen fixation, which was previously recognized in *Azoarcus*, *Azonexus*, *Azospira* and *Azovibrio*^54^, was found widely distributed in many groups. For example, all *Uliginosibacterium_f* strains have complete nitrogen fixation genes in their genomes, whereas this feature has not been experimentally validated in these organisms^56^.

**Table 1:** Distribution of genes related to key functions of certain taxa in *Rhodocyclales*.

Function	Genes or enzymes	Distribution
Anoxygenic photoheterotrophy	*pufL*, *pufM*	Only present in *R. tenuis*
Nitrogen fixation	*nifA*, *nifB*, *nifL*, *nifD*, *nifK*, *nifH*	All *Uliginosibacterium_f* Around half in *Azonexaceae* and *Rhodocyclaceae* A few in *Zoogloeaceae*; One of *Azospira_f* (*Azospira oryzae*, *nifL* missing)
Chlorate respiration	Chlorate reductase	Two in *Zoogloeaceae* (DFZZ01, EbN1)
Perchlorate respiration	Perchlorate reductase	Two in *Azonexaceae* (*D. hortensis*, *F. limneticum*) One in *Azospira_f* (*Azospira oryzae*) Five in *Zoogloeaceae* (*Azo*. t*oluclasticus*, EbN1, *T. phenylacetica*, MZ1T, DFZZ01)
Selenate respiration	Selenate reductase	Three in *Zoogloeaceae* (EbN1, *Azo. toluclasticus*, *T. terpenica*)
ROS detoxification	Superoxide dismutase, Catalase-peroxidase	Nearly all genomes

Respiration on chlorate or perchlorate was previously discovered in strains from *Azospira*, *Dechloromonas* and *Propionivibrio*, whereas selenate could serve as electron acceptor only for *T*. *selenatisa*^54^. Interestingly, the strain of *D. agita* has been validated in chlorate respiration^57^, but the functional gene was absent in the complete genome of the non-type strain of the same species, that is, JAET01. Two strains in *Zoogloeaceae* contained chlorate reductase and eight strains, two from *Azonexaceae*, one from *Azospira_f* and five from *Zoogloeaceae* carried gene-encoding perchlorate reductase. Only *A. oryzae* and *D. hortensis* were experimentally verified^54^. The genome EbN1, which is an invalid taxon *Aromatoleum aromaticum* previously^58^, surprisingly contained all three reductases. Therefore, the versatility of this genome on diverse electron acceptors was evident. Our survey based on gene content greatly expanded the strain spectrum that could respire on these electron acceptors. However, a patchy distribution of these genes among families, genera and even within species suggested that they were unsuitable for taxonomic basis.

### Genomics revealed distribution and potential horizontal transfer of denitrifying genes in *Rhodocyclales*

As shown in Fig. 2, *narG* was detected in all families except for *Azospira_f*, whereas *nirS* was completely absent only in *Uliginosibacterium_f*. However, *narG* exhibited a patchy pattern, but *nirS* was widely present in most genomes in the four families. *nirK* was only found in the three genomes from *Ca*. Dactylopiibacterium carminicum. *nirK* and *nirS* are usually mutually exclusive in most cases, although *nir* type may differ within closely related taxa^21,59^. *norB* and *nosZ* showed a co-occurred tendency in most lineages, except for many *Rhodocyclaceae* genomes, which were absent in *norB* and present in *nosZ*, as reported by a previous summary on *Accumulibacter*^60^. Two strains, namely, *A. restrictus* DSM23866 and XMAS_Bin103, containing *norB* but not *nosZ*, might possibly produce nitrous oxide during denitrifying^61^. However, the absence of *nosZ* in the latter might be derived from the low genomic completeness (<75%, Table S1). The previous survey on the co-occurrence of denitrifying genes has shown that strains containing *nirS*, instead of *nirK*, are more likely to include all downstream denitrifying genes^62^. This conclusion could be generally supported by the majority of examined *Rhodocyclales* genomes except for *Rhodocyclaceae*. The *nirS*-containing genomes in this order usually contain the downstream genes of *norB* and *nosZ*.

The co-existence of multiple copies of denitrification genes in certain genomes has been observed, especially for *nirS*^63,64^. Multiple copies of *NIR* genes might be related to alternative functions, niche adaption and different selective pressures due to HGT or gene duplication^65^. Fitness advantages of multiple copies of *nirS* have been experimentally validated on *Thauera* spp.^66^. In the *Rhodocyclales* genomes, the ORFs of *narG*, *nirS*, *norB* and *nosZ* could be divided into nine orthologous clusters (designated as NarG1, NarG2, NirS1, NirS2, NirS3, NorB1, NorB2, NosZ1 and NosZ2, Table S3). More than one copy of each gene was detected in many *Rhodocyclales* genomes (Fig. 2 and Table S3). The multicopy of *nirS* could be divided into two types. The first was that the copies were within the same gene cluster; thus, they were likely to be generated from gene duplication or gained from closely related taxa. The other type consisted of copies with large divergence (not grouped in a gene cluster). The complex and patchy distribution of multicopy *nirS* (Fig. 2 and Table S3) suggested frequent gene loss or horizontal gain gene from related taxa during diversifying.

On the one hand, we constructed the phylogenetic tree of NirS covering popular denitrifying bacterial lineages (Fig. S3) to track the potential horizontal transfer events of denitrifying ORFs between *Rhodocyclales* and other taxa. In the tree of six clades (clades I–VI), *nirS* sequences from *Rhodocyclales* dispersedly located in three clades (clades III, V and VI), while they were absent from two root clades (clades I and II). One clade exclusively comprised Gamma- and Alpha-proteobacteria lineages (clade IV). ORFs in the three clades corresponded with the three NirS clusters (NirS1, NirS2 and NirS3). Noticeably, in at least two clades (clades V and VI) that contained taxa from multiple bacterial classes, *Rhodocyclales* sequences located in the root. Therefore, they or their ancestor taxa might be the donors of horizontal transfer events before the subsequent evolution of the clades. Although a previous study has proposed that *nirS* is overall congruent with rRNA-based phylogeny with occasional horizontal transfer events^65^, our results suggested that the events might be frequently detected if additional genomes were presented.

**Figure 3:**
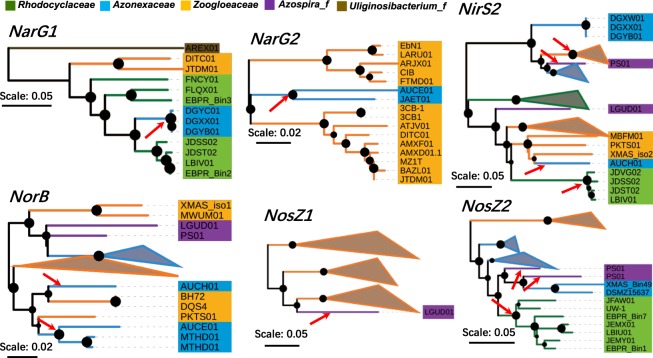
Neighbor-joining trees of denitrifying gene clusters of *Rhodocyclales*. Six of the seven gene clusters were present with signs of horizontal transfer events. Arrows show the potential horizontal transfer events, where a branch was inserted to a certain lineage of another family. The nodes with BS value over 50% (n = 1000) were present and the size increased with the value.

On the other hand, we further profiled potential within-order HGT events for each of the seven denitrifying gene clusters (NirS3 and NorB2 were excluded due to the small number of sequences). As shown in Fig. 3, except for NirS1, all the six other clusters had supporting evidence of interfamily HGT events. Strong evidence of a single HGT event was observed in NarG1, NarG2 and NosZ1, the former two of which were supported by multiple genomes from one family. Thus, their sequences formed a monophyletic branch, which was inserted to a lineage of another family. Multiple events were observed in NirS2, NorB1 and NosZ2, where the true transferring history might be difficult to track. The genomes of DGXX01, DGYB01, AUCE01, AUCH01, LGUD01 and *A. oryzae* PS exhibited horizontal transfer events in multiple clusters. Given that all these genomes were generated from isolates, the unusual phylogeny might not be derived from genome contamination. As pointed out by Soucy *et al*.^67^, HGT between closely related organisms may occur frequently but is difficult to detect owing to the indistinguishable incongruence between the HGT gene and phylogeny. However, the inter-family HGT of the denitrifying gene was detectable and did not occasionally occur within *Rhodocyclales*. This result indicated that several taxa in the order could gain (or restore) the denitrification capability by acquiring functional genes from the related taxa in the order, which conferred certain superiority in their ecological adaption. A closely related source of HGT gene might have potential merits in functionality and fitness^68^.

### Distribution of major sub-lineages of *Rhodocyclales* in global WWTPs were significantly governed by temperature and MLSS

**Figure 4:**
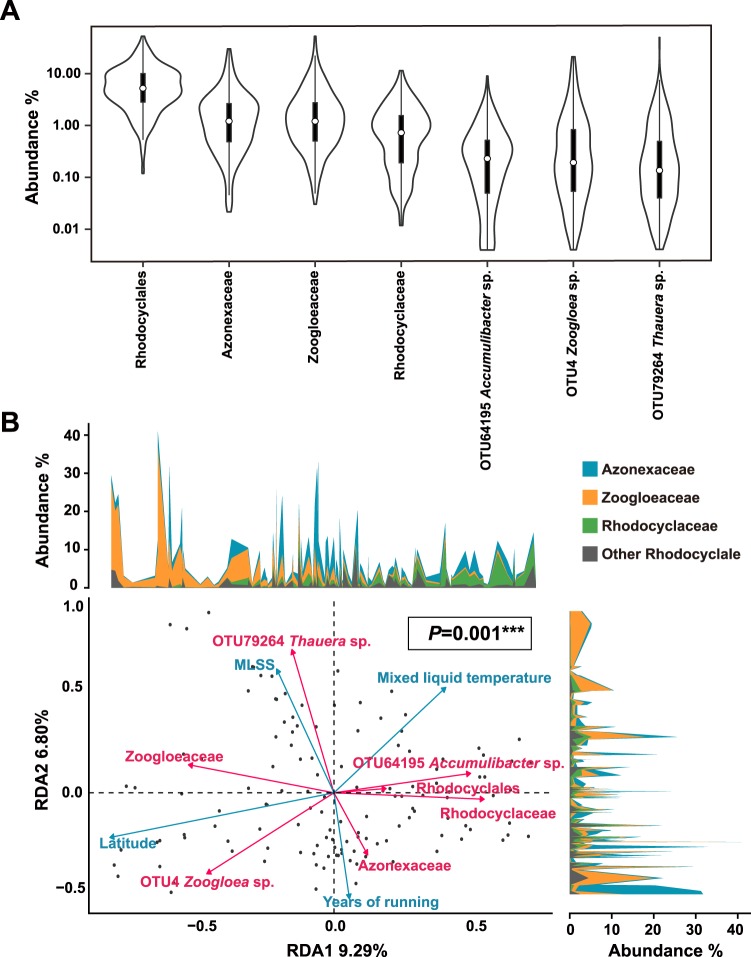
Distribution of *Rhodocyclales* and the factors governing its distribution in WWTPs. (**A**) Violin plot showing the abundance distribution of *Rhodocyclales* in global WWTPs. Only taxa with the median abundance of >0.1% are shown. (**B**) RDA plot based on *Rhodocyclales* across 131 WWTPs. The *P* value shown in the plot was estimated by anova.cca function and each circle represents a WWTP. The red arrows indicate the taxa and the blue arrows indicate the environmental variables with *P* < 0.05 (envfit function). The top and right panels show the abundance distribution of the *Rhodocyclales* at the family level along with the first and second constrained axes.

As shown in Fig. 4A, *Rhodocyclales* accounted for 7.7% of the total bacterial community in WWTPs. This finding was generally consistent with another survey at the global level with few WWTPs^5^. The three families, namely, *Azonexaceae*, *Rhodocyclaceae* and *Zoogloeaceae*, accounted for 2.9%, 1.1% and 2.6% on average, respectively. Three top OTUs, namely, *Accumulibacter* sp., *Zoogloea* sp. and *Thauera* sp., accounted for 0.3%, 1.1% and 0.4% on average, respectively.

The RDA analysis (Fig. 4B) showed that the abundances of *Rhodocyclales*, *Rhodocylaceae* and *Accumulibacter* OTU were negatively correlated to the latitude and positively correlated to temperature in full-scale WWTPs^24^. Previous reports on EBPR have shown optimal phosphate removal performance at 10 °C to 20 °C^69,70^, but these studies have focused on the competition relationship between polyphosphate- and glycogen-accumulating organisms in laboratory EBPR systems with enriched *Accumulibacter*. Except for one study on 18 WWTPs by Mao *et al*.^71^, information on factors governing the distribution of the aforementioned genus in considerably low abundance in full-scale WWTPs is scarce. Our result suggested that the distribution of *Accumulibacter* could be selected with high temperature under the subdominant situation in full-scale WWTPs, which was similar to that of Mao *et al*.^71^.

*Zoogloeaceae* is negatively correlated with *Rhodocylaceae* and dominates in low-temperature areas. Currently, no other evidence supports the low temperature adaptive growth advantage of *Zoogloeaceae*. The production of extracellular polysaccharides (EPSs) in sewage treatment is negatively correlated with temperature^72^. The advantage of *Zoogloea* at low temperatures might be related to the increase in EPS production. A possible explanation is that most microorganisms grow relative slowly at low ambient temperature, while the aggregates formed by *Zoogloea*^73^ can retain in AS biomass via minimizing the elution process compared with other taxa.

Interestingly, *Azonexaceae* was positively correlated with the running years of the WWTP and negatively correlated with MLSS. Low MLSS indicated high efficiency in treatment when the operational load and performance were comparable. Therefore, *Azonexaceae* might be associated with high treating efficiency in wastewater treatment.

### Metagenomics suggested predominant role of *Rhodocyclales* in denitrifying in wastewater treatment systems

**Figure 5:**
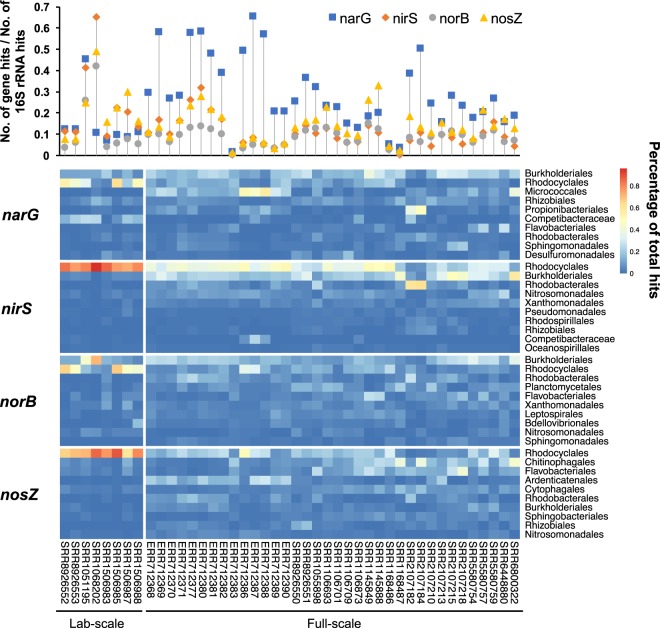
Distribution of four denitrifying genes in 44 activated sludge metagenomes. Dropline diagram on the topside shows the normalized abundance of each 16S rRNA gene. For each gene, the relative abundances of the top 10 orders (sorted by the mean value of relative abundance for each gene) are displayed in the heatmap.

Although previous rRNA-based investigations have shown that *Rhodocyclales* is a top lineage in AS samples^5,74^, its contribution to the denitrifying gene pool has not been comprehensively characterized. We taxonomically quantified *narG*, *nirS*, *norB* and *nosZ* in metagenomic data from 44 globally collected AS samples. As shown in Fig. 5, *narG* was the most abundant in most samples compared with the three other genes because of its long length. SRR1068202, which is an AS sample from a laboratory EBPR bioreactor, showed a very high abundance of *nirS* and a low abundance of *narG*. We speculated that the dominant taxa in the system should be *Accumulibacter* population without *narG*.

*Rhodocyclales*-derived denitrifying genes were all ranked at the top or the second among all orders. Therefore, they significantly contributed to the denitrifying process. Eight laboratory reactors were likely dominated by this order based on the datasets possibly due to a biased sampling from EBPR systems. For 36 full-scale samples, *Rhodocyclales*-derived *nirS* and *nosZ* were ranked as the top in 33 and 21 samples, respectively, whereas the numbers were 9 and 10 for *narG* and *norB*, respectively. Another order in *Betaproteobacteria*, that is, *Burkholderiales*, was also an important contributor to potential denitrifiers because it ranked as the top for *narG* and *norB*, followed by *nirS*. Its abundance was close to that of *Rhodocyclales* according to a 16S rRNA survey on multiple globally collected AS samples^5,74^. However, this order only slightly contributed to *nosZ* (ranked at the seventh order).

Metagenomic survey on a few AS samples has confirmed the contribution of *Rhodocyclales* in the denitrifying community^19^. Surprisingly, no comprehensive PCR-based survey on the diversity of denitrifiers in AS has been performed. A recent study performed by Ma *et al*.^75^ updated the primers for detecting environmental denitrifiers, which would facilitate the study of denitrifier diversity in AS samples together with the metagenomic approach, as applied by the present study. Further combination of transcriptional and biochemical studies on the denitrifying process in full-scale AS is important to understand the true functional contribution and operational parameters related to their functions.

## Conclusions

On the basis of the information of 78 *Rhodocyclales* genomes and metagenomes from wastewater treatment systems, we provided novel insights into the phylogeny, denitrification and the distribution in WWTPs of this bacterial order. First, *Rhodocyclales* could at least be divided into five family-level lineages, while two of them contained limited genomes currently. This finding should be further validated with more genomes available. Second, evidence of multicopy and within-order horizontal transfer of denitrifying genes was observed widely. Meanwhile, this order was the major contributor to the denitrifying gene pools in AS samples as revealed by metagenomics. Further study on the expression-level was important to confirm their functional contribution in AS. Third, the most important governing variables for different families of *Rhodocyclales* in global WWTPs were temperature and MLSS. Their function and niche adaption at a more detailed taxonomic level (e.g., genus or species) in AS should be further investigated comprehensively as more available metadata and genomes can be retrieved.

## Supplementary information

Supplementary Figures.

Supplementary tables.

## References

[1] Sheik AR, Muller EEL, Wilmes P (2014). A hundred years of activated sludge: Time for a rethink. Front. Microbiol.

[2] Van Loosdrecht MCM, Brdjanovic D (2014). Anticipating the next century of wastewater treatment. Science.

[3] Henze, M. van,L. M., E. G., B. D. Biological Wastewater Treatment. In Chemical Engineering 2–9 (CRC Press, 2011).

[4] Loy A (2005). 16S rRNA gene-based oligonucleotide microarray for environmental monitoring of the betaproteobacterial order ‘ *Rhodocyclales*’. Appl. Environ. Microbiol..

[5] Zhang T, Shao MF, Ye L (2012). 454 Pyrosequencing reveals bacterial diversity of activated sludge from 14 sewage treatment plants. ISME J..

[6] Hesselmann RPX, Werlen C, Hahn D, Van Der Meer JR, Zehnder AJB (1999). Enrichment, phylogenetic analysis and detection of a bacterium that performs enhanced biological phosphate removal in activated sludge. Syst. Appl. Microbiol..

[7] Shin YK, Hiraishi A, Sugiyama J (1993). Molecular systematics of the genus *Zoogloea* and emendation of the genus. Int. J. Syst. Bacteriol..

[8] Anders HJ, Kaetzke A, Kampfer P, Ludwig W, Fuchs G (1995). Taxonomic position of aromatic-degrading denitrifying pseudomonad strains K 172 and KB 740 and their description as new members of the genera *Thauera*, as *Thauera aromatica* sp. nov., and *Azoarcus*, as *Azoarcus evansii* sp. nov., respectively, members of the beta subclass of the *Proteobacteria*. Int. J. Syst. Bacteriol..

[9] Mechichi T, Stackebrandt E, Gad’on N, Fuchs G (2002). Phylogenetic and metabolic diversity of bacteria degrading aromatic compounds under denitrifying conditions, and description of *Thauera phenylacetica* sp. nov., *Thauera aminoaromatica* sp. nov., and *Azoarcus buckelii* sp. nov. Arch. Microbiol..

[10] Ginige MP, Keller J, Blackall LL (2005). Investigation of an acetate-fed denitrifying microbial community by stable isotope probing, full-cycle rRNA analysis, and fluorescent *in situ* hybridization-microautoradiography. Appl. Environ. Microbiol..

[11] Thomsen TR, Kong Y, Nielsen PH (2007). Ecophysiology of abundant denitrifying bacteria in activated sludge. FEMS Microbiol. Ecol.

[12] Morgan-Sagastume F, Nielsen JL, Nielsen PH (2008). Substrate-dependent denitrification of abundant probe-defined denitrifying bacteria in activated sludge. FEMS Microbiol. Ecol.

[13] Aladame, N. Bergey’s Manual® of Systematic Bacteriology. Springer, New York, NY. **138** (2005).

[14] Boden R, Hutt LP, Rae AW (2017). Reclassification of *Thiobacillus aquaesulis* (Wood & Kelly, 1995) as *Annwoodia aquaesulis* gen. nov., comb. nov., transfer of *Thiobacillus* (Beijerinck, 1904) from the *Hydrogenophilales* to the *Nitrosomonadales*, proposal of *Hydrogenophilalia* class. nov. within the ‘*Proteobacteria*’, and four new families within the orders *Nitrosomonadales* and *Rhodocyclales*. Int. J. Syst. Evol. Microbiol..

[15] Naushad S (2015). A phylogenomic and molecular marker based taxonomic framework for the order *Xanthomonadales*: proposal to transfer the families *Algiphilaceae* and *Solimonadaceae* to the order Nevskiales ord. nov. and to create a new family within the order Xanthomonadales. Antonie van Leeuwenhoek, Int. J. Gen. Mol. Microbiol..

[16] Hug, L. A. *et al*. A new view of the tree of life. *Nat. Microbiol*. **1** (2016). 10.1038/nmicrobiol.2016.48 27572647

[17] Simon M (2017). Phylogenomics of *Rhodobacteraceae* reveals evolutionary adaptation to marine and non-marine habitats. ISME J..

[18] Srinandan CS, Shah M, Patel B, Nerurkar AS (2011). Assessment of denitrifying bacterial composition in activated sludge. Bioresour. Technol.

[19] Wang Z (2014). Abundance and diversity of bacterial nitrifiers and denitrifiers and their functional genes in tannery wastewater treatment plants revealed by highthroughput sequencing. PLoS One.

[20] Heylen K (2007). Nitric oxide reductase (*norB*) gene sequence analysis reveals discrepancies with nitrite reductase (*nir*) gene phylogeny in cultivated denitrifiers. Environ. Microbiol..

[21] Jones CM, Hallin S (2010). Ecological and evolutionary factors underlying global and local assembly of denitrifier communities. ISME J..

[22] Clays-Josserand A, Ghiglione JF, Philippot L, Lemanceau P, Lensi R (1999). Effect of soil type and plant species on the fluorescent pseudomonads nitrate dissimilating community. Plant Soil.

[23] Philippot L (2002). Denitrifying genes in bacterial and Archaeal genomes. Biochimica et Biophysica Acta - Gene Structure and Expression.

[24] Wu L (2019). Global diversity and biogeography of bacterial communities in wastewater treatment plants. Nat. Microbiol.

[25] Yoon SH (2017). Introducing EzBioCloud: A taxonomically united database of 16S rRNA gene sequences and whole-genome assemblies. Int. J. Syst. Evol. Microbiol.

[26] Lagesen K (2007). RNAmmer: Consistent and rapid annotation of ribosomal RNA genes. Nucleic Acids Res.

[27] Bankevich A (2012). SPAdes: a new genome assembly algorithm and its applications to single-cell sequencing. J. Comput. Biol..

[28] Li D, Liu CM, Luo R, Sadakane K, Lam TW (2015). MEGAHIT: An ultra-fast single-node solution for large and complex metagenomics assembly via succinct de Bruijn graph. Bioinformatics.

[29] Albertsen M (2013). Genome sequences of rare, uncultured bacteria obtained by differential coverage binning of multiple metagenomes. Nat. Biotechnol..

[30] Karst, S. M., Kirkegaard, R. H. & Albertsen, M. Mmgenome: a toolbox for reproducible genome extraction from metagenomes. *bioRxiv**059121*, 10.1101/059121 (2014).

[31] Langmead B, Salzberg SL (2012). Fast gapped-read alignment with Bowtie 2. Nat. Methods.

[32] Li H (2009). The Sequence Alignment/Map format and SAMtools. Bioinformatics.

[33] Parks DH, Imelfort M, Skennerton CT, Hugenholtz P, Tyson GW (2015). CheckM: assessing the quality of microbial genomes recovered from isolates, single cells, and metagenomes. Genome Res..

[34] Katoh K, Standley DM (2013). MAFFT multiple sequence alignment software version 7: improvements in performance and usability. Mol. Biol. Evol..

[35] Kumar S, Stecher G, Tamura K (2016). MEGA7: Molecular evolutionary genetics analysis version 7.0 for bigger datasets. Mol. Biol. Evol..

[36] Stamatakis A (2014). RAxML version 8: A tool for phylogenetic analysis and post-analysis of large phylogenies. Bioinformatics.

[37] Ronquist F, Huelsenbeck JP (2003). MrBayes 3: Bayesian phylogenetic inference under mixed models. Bioinformatics.

[38] Wu D, Jospin G, Eisen JA (2013). Systematic identification of gene families for use as ‘markers’ for phylogenetic and phylogeny-driven ecological studies of bacteria and archaea and their major subgroups. PLoS One.

[39] Castresana J (2000). Selection of conserved blocks from multiple alignments for their use in phylogenetic analysis. Mol. Biol. Evol.

[40] Seemann T (2014). Prokka: Rapid prokaryotic genome annotation. Bioinformatics.

[41] McGill SE, Barker D (2017). Comparison of the protein-coding genomes of three deep-sea, sulfur-oxidising bacteria: ‘ *Candidatus* Ruthia magnifica’, ‘*Candidatus* Vesicomyosocius okutanii’ and *Thiomicrospira crunogena*. BMC Res. Notes.

[42] Schloss PD (2009). Introducing mothur: Open-source, platform-independent, community-supported software for describing and comparing microbial communities. Appl. Environ. Microbiol..

[43] Oksanen J (2017). Community Ecology Package ‘vegan’. Version 2.4-3. R Packag..

[44] Schuler Gregory D., Epstein Jonathan A., Ohkawa Hitomi, Kans Jonathan A. (1996). [10] Entrez: Molecular biology database and retrieval system. Methods in Enzymology.

[45] Jensen L. J., Julien P., Kuhn M., von Mering C., Muller J., Doerks T., Bork P. (2007). eggNOG: automated construction and annotation of orthologous groups of genes. Nucleic Acids Research.

[46] Buchfink B, Xie C, Huson DH (2014). Fast and sensitive protein alignment using DIAMOND. Nat. Methods.

[47] Eddy SR (1998). Profile hidden Markov models. Bioinformatics.

[48] Oyserman BO (2016). Ancestral genome reconstruction identifies the evolutionary basis for trait acquisition in polyphosphate accumulating bacteria. ISME J..

[49] Wilke A (2016). The MG-RAST metagenomics database and portal in 2015. Nucleic Acids Res..

[50] Kopylova E, Noé L, Touzet H (2012). SortMeRNA: Fast and accurate filtering of ribosomal RNAs in metatranscriptomic data. Bioinformatics.

[51] Kolde R (2015). pheatmap: Pretty Heatmaps. R Packag. version.

[52] Ludwig Wolfgang, Euzéby Jean, Schumann Peter, Busse Hans-Jürgen, Trujillo Martha E., Kämpfer Peter, Whitman William B. (2012). Road map of the phylum Actinobacteria. Bergey’s Manual® of Systematic Bacteriology.

[53] Whitman WB (2015). Genome sequences as the type material for taxonomic descriptions of prokaryotes. Systematic and Applied Microbiology.

[54] Oren Aharon (2014). The Family Rhodocyclaceae. The Prokaryotes.

[55] McCord JM, Keele BB, Fridovich I (1971). An enzyme-based theory of obligate anaerobiosis: The physiological function of superoxide dismutase. Proc. Natl. Acad. Sci..

[56] Weon HY (2008). *Uliginosibacterium gangwonense* gen. nov., sp. nov. isolated from a wetland, Yongneup, in Korea. Int. J. Syst. Evol. Microbiol..

[57] Achenbach LA, Michaelidou U, Bruce RA, Fryman J, Coates JD (2001). *Dechloromonas agitata* gen. nov., sp. nov. and *Dechlorosoma suillum* gen. nov., sp. nov., two novel environmentally dominant (per)chlorate-reducing bacteria and their phylogenetic position. Int. J. Syst. Evol. Microbiol.

[58] Rabus R, Widdel F (1995). Anaerobic degradation of ethylbenzene and other aromatic hydrocarbons by new denitrifying bacteria. Arch. Microbiol..

[59] Coyne MS, Arunakumari A, Averill BA, Tiedje JM (1989). Immunological identification and distribution of dissimilatory heme cd1 and nonheme copper nitrite reductases in denitrifying bacteria. Appl. Environ. Microbiol..

[60] Camejo PY, Oyserman BO, McMahon KD, Noguera DR (2019). Integrated omic analyses provide evidence that a “ *Candidatus* Accumulibacter phosphatis” strain performs denitrification under microaerobic conditions. mSystems.

[61] Philippot L, Andert J, Jones CM, Bru D, Hallin S (2011). Importance of denitrifiers lacking the genes encoding the nitrous oxide reductase for N O emissions from soil. Glob. Chang. Biol.

[62] Graf DRH, Jones CM, Hallin S (2014). Intergenomic comparisons highlight modularity of the denitrification pathway and underpin the importance of community structure for N O emissions. PLoS One.

[63] Fukumori, Y., Oyanagi, H., Yoshimatsu, K., Noguchi, Y. & Fujiwara, T. Enzymatic iron oxidation and reduction in magnetite synthesizing Magnetospirillum magnetotacticum. J. Phys. IV JP 7, C1-659-C1-662 (1997).

[64] Rinaldo Serena, Cutruzzolà Francesca (2007). Nitrite Reductases in Denitrification. Biology of the Nitrogen Cycle.

[65] Jones CM, Stres B, Rosenquist M, Hallin S (2008). Phylogenetic analysis of nitrite, nitric oxide, and nitrous oxide respiratory enzymes reveal a complex evolutionary history for denitrification. Mol. Biol. Evol.

[66] Etchebehere C, Tiedje J (2005). Presence of two different active nirS nitrite reductase genes in a denitrifying *Thauera* sp. from a high-nitrate-removal-rate reactor. Appl. Environ. Microbiol..

[67] Soucy SM, Huang J, Gogarten JP (2015). Horizontal gene transfer: Building the web of life. Nat. Rev. Genet..

[68] Porse, A., Schou, T. S., Munck, C., Ellabaan, M. M. H. & Sommer, M. O. A. Biochemical mechanisms determine the functional compatibility of heterologous genes. *Nat. Commun*. **9** (2018). 10.1038/s41467-018-02944-3 PMC5802803 29410400

[69] Panswad T, Doungchai A, Anotai J (2003). Temperature effect on microbial community of enhanced biological phosphorus removal system. Water Res.

[70] Lopez-Vazquez, C. M. The competition between polyphosphate-accumulating organisms and glycogen-accumulating organisms: temperature effects and modelling. *CRC Press* (2009).

[71] Mao, Y., Graham, D. W., Tamaki, H. & Zhang, T. Dominant and novel clades of *Candidatus* Accumulibacter phosphatis in 18 globally distributed full-scale wastewater treatment plants. *Sci. Rep*. **5** (2015). 10.1038/srep11857 PMC4490554 26138542

[72] Wang Z, Wu Z, Tang S (2009). Extracellular polymeric substances (EPS) properties and their effects on membrane fouling in a submerged membrane bioreactor. Water Res.

[73] Pike EB, Curds CR (1971). The microbial ecology of the activated sludge process. Society for Applied Bacteriology symposium series.

[74] Hu M, Wang X, Wen X, Xia Y (2012). Microbial community structures in different wastewater treatment plants as revealed by 454-pyrosequencing analysis. Bioresour. Technol.

[75] Ma Y, Zilles JL, Kent AD (2019). An evaluation of primers for detecting denitrifiers via their functional genes. Environ. Microbiol..

