# Supplementary material for: Phylogenomics of Rhodocyclales and its distribution in wastewater treatment systems
Source: Sci Rep. 2020 Mar 3;10:3883. doi: 10.1038/s41598-020-60723-x (PMC7054561; doi:10.1038/s41598-020-60723-x)
Supplement: Supplementary file 1 — Supplementary Figures. [file 41598_2020_60723_MOESM1_ESM.pdf]

Supplementary Figures for

Phylogenomics of *Rhodocyclales* and its distribution in wastewater treatment systems

Zhongjie Wang[1], Wenqing Li[2], Hao Li[1], Wei Zheng[1], Feng Guo[*,1]

1. School of Life Sciences, Xiamen University, Fujian 361102, PR China
2. College of The Environments and Ecology, Xiamen University, Fujian 361102, PR China

[*]Corresponding author

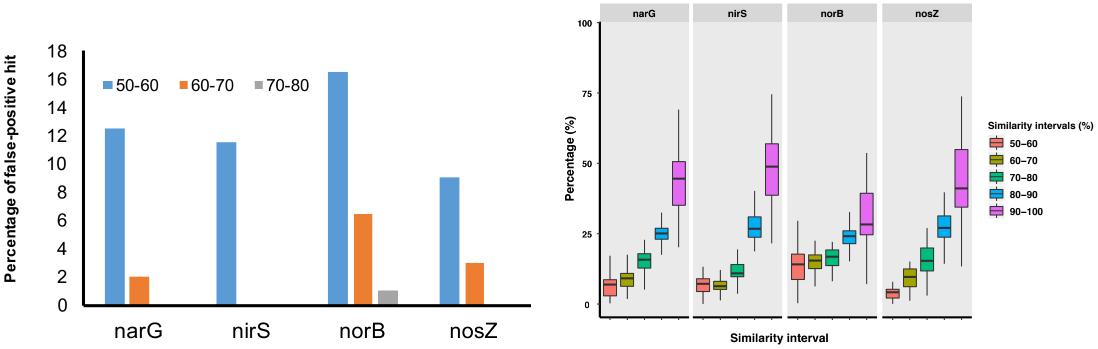

Figure S1. False-positive rates of denitrifying genes (A) and distribution of hits between metagenomic reads and the denitrifying gene references (B) under various similarity cutoffs. The similarity cutoff at 70% was used in the final dataset because the low false positive frequency and low abundance of hits below the cutoff.

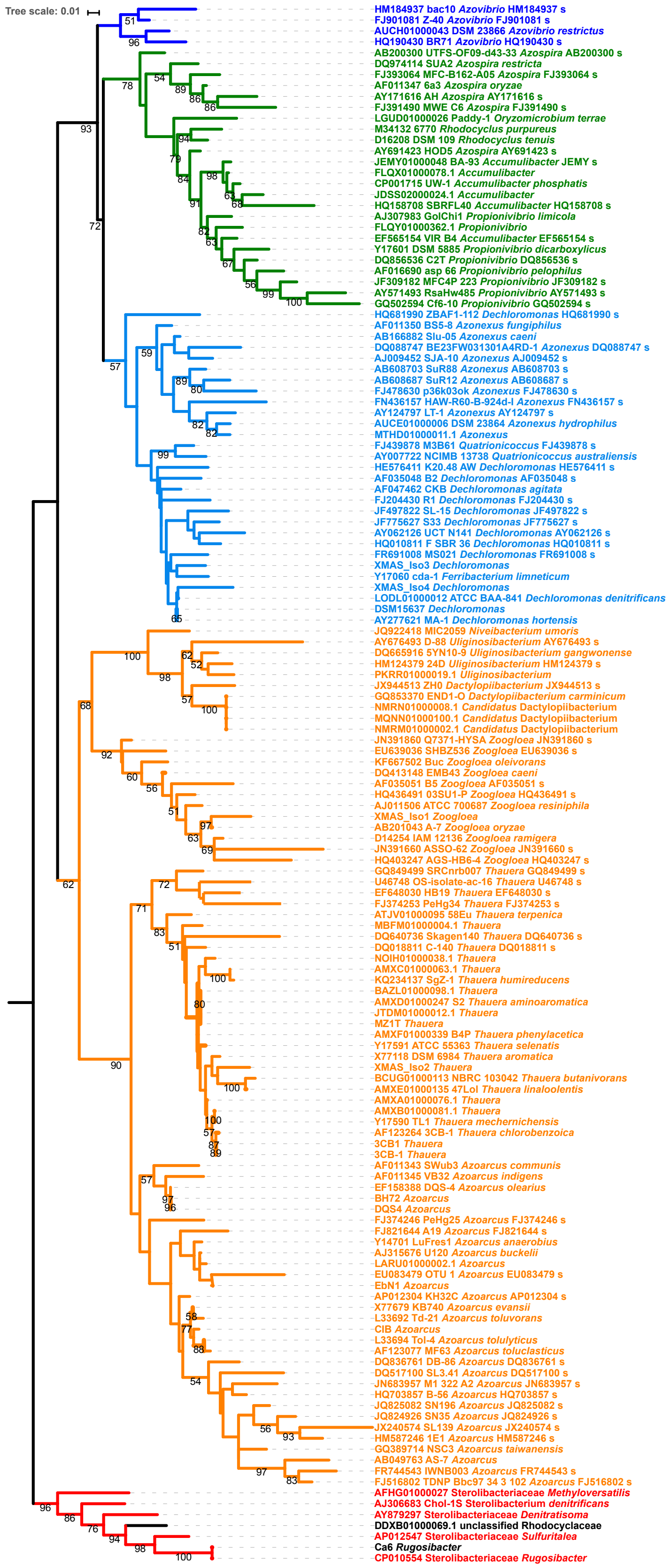

Figure S2. Full ML tree on the basis of the 16S rRNA gene of the order *Rhodocyclales*.

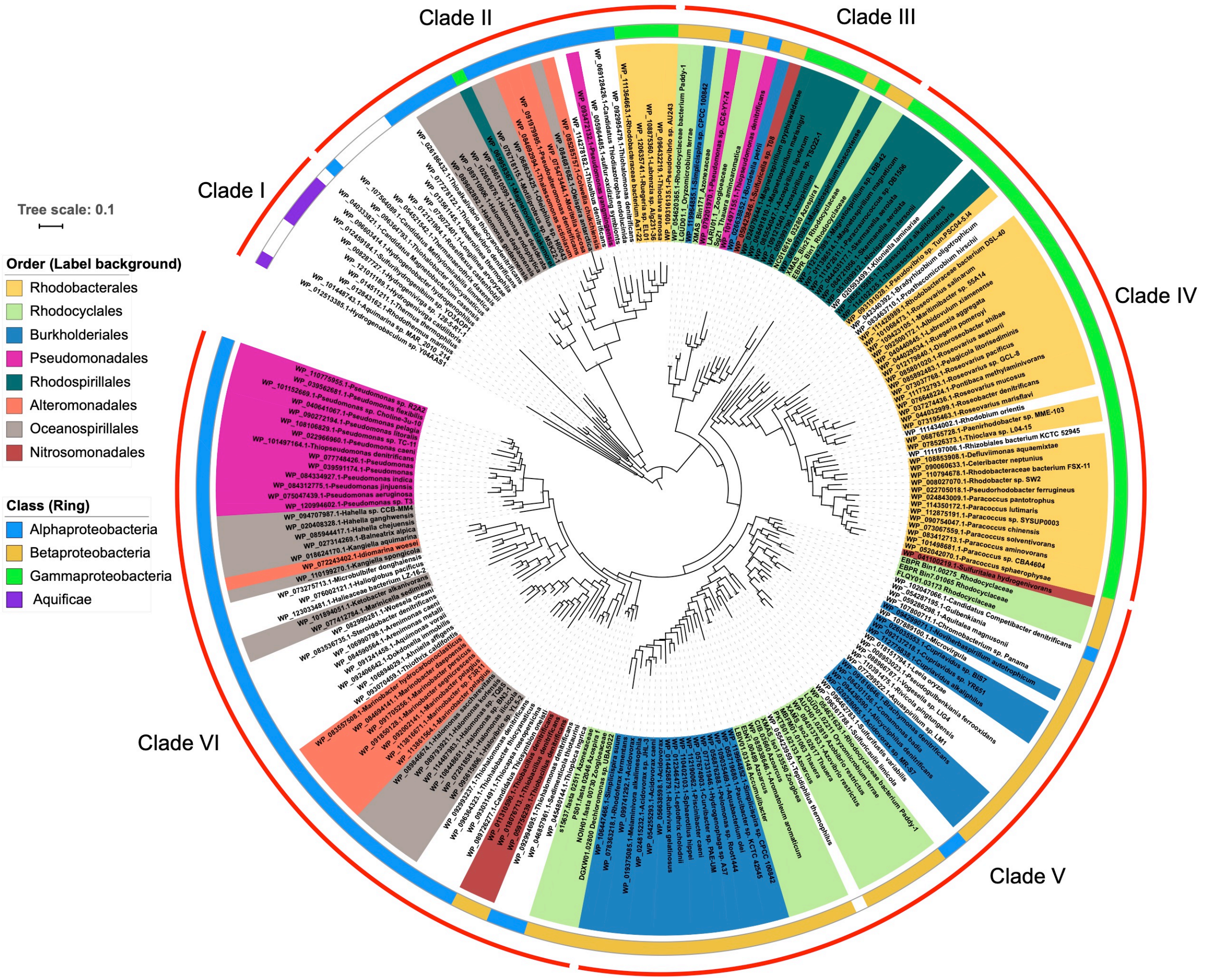

Figure S3. Phylogenetic tree on the basis of from 217 representative *nirS* sequences.
